# The Multiple Facets of Plant–Fungal Interactions Revealed Through Plant and Fungal Secretomics

**DOI:** 10.3389/fpls.2019.01626

**Published:** 2020-01-08

**Authors:** Delphine Vincent, Maryam Rafiqi, Dominique Job

**Affiliations:** ^1^ Agriculture Victoria Research, AgriBio, Centre for AgriBioscience, Bundoora, VIC, Australia; ^2^ AgroBioSciences Program, Mohammed VI Polytechnic University (UM6P), Ben Guerir, Morocco; ^3^ CNRS/Université Claude Bernard Lyon 1/Institut National des Sciences Appliquées/Bayer CropScience Joint Laboratory (UMR 5240), Bayer CropScience, Lyon, France

**Keywords:** agriculture, apoplast, extracellular vesicles, fungal effectors, plant–fungi interactions, root exudates, whole and integrated secretomics

## Abstract

The plant secretome is usually considered in the frame of proteomics, aiming at characterizing extracellular proteins, their biological roles and the mechanisms accounting for their secretion in the extracellular space. In this review, we aim to highlight recent results pertaining to secretion through the conventional and unconventional protein secretion pathways notably those involving plant exosomes or extracellular vesicles. Furthermore, plants are well known to actively secrete a large array of different molecules from polymers (e.g. extracellular RNA and DNA) to small compounds (e.g. ATP, phytochemicals, secondary metabolites, phytohormones). All of these play pivotal roles in plant-fungi (or oomycetes) interactions, both for beneficial (mycorrhizal fungi) and deleterious outcomes (pathogens) for the plant. For instance, recent work reveals that such secretion of small molecules by roots is of paramount importance to sculpt the rhizospheric microbiota. Our aim in this review is to extend the definition of the plant and fungal secretomes to a broader sense to better understand the functioning of the plant/microorganisms holobiont. Fundamental perspectives will be brought to light along with the novel tools that should support establishing an environment-friendly and sustainable agriculture.

## Introduction

Plants and fungi secrete a wide range of molecules into the extracellular space, where they play crucial roles in signaling, development and stress responses (Delaunois et al., 2014). Proteins constitute the most intensively studied group of these molecules. Until recently, current paradigm in plant-microbe interactions suggested that secreted proteins are synthesized and delivered through the conventional endoplasmic reticulum (ER) secretory pathway, which is based on the specific recognition of N-terminally located transit peptides (Agrawal et al., 2010; Giraldo et al., 2013). However, recent work disclosed the secretion of a new type of secreted proteins, devoid of transit peptide, referred to as leaderless secretory proteins (LSPs) (Wang et al., 2017), supporting the existence of novel secretory mechanisms independent of the classical ER-Golgi pathway. These unconventional secretion pathways involve small extracellular vesicles (EVs) for the export of proteins to the extracellular milieu and have recently been shown to mediate plant defense against invasive fungal pathogens. Indeed, it has been observed that when an invasive fungal pathogen takes up plant EVs, its growth is inhibited as a direct consequence of plant EVs absorption (Regente et al., 2017).

EVs, including exosomes, are small, membrane-enclosed structures released from a cell into the surrounding environment. They play a pivotal role in cell-to-cell communications and host-pathogen interactions, through the transport and exchange of molecules. Ubiquitous, EVs are found in prokaryotes and eukaryotes, as well as unicellular and multicellular organisms (Yanez-Mo et al., 2015). To date, several studies have mainly focused on human EVs for their potential applications in medicine, underpinned by the development of EV biomarkers for diagnostic and therapeutic tools (Bielska et al., 2019). The idea that organisms possessing strong cellular physical barriers such as cell walls (CWs), like plants and fungi, could produce entities as large as EVs that make it past the CW has seemed questionable, albeit is now a fact (Casadevall et al., 2009; Brown et al., 2015; Bleackley et al., 2019). How this phenomenon eventuates remains to be elucidated.

**Figure 1:**
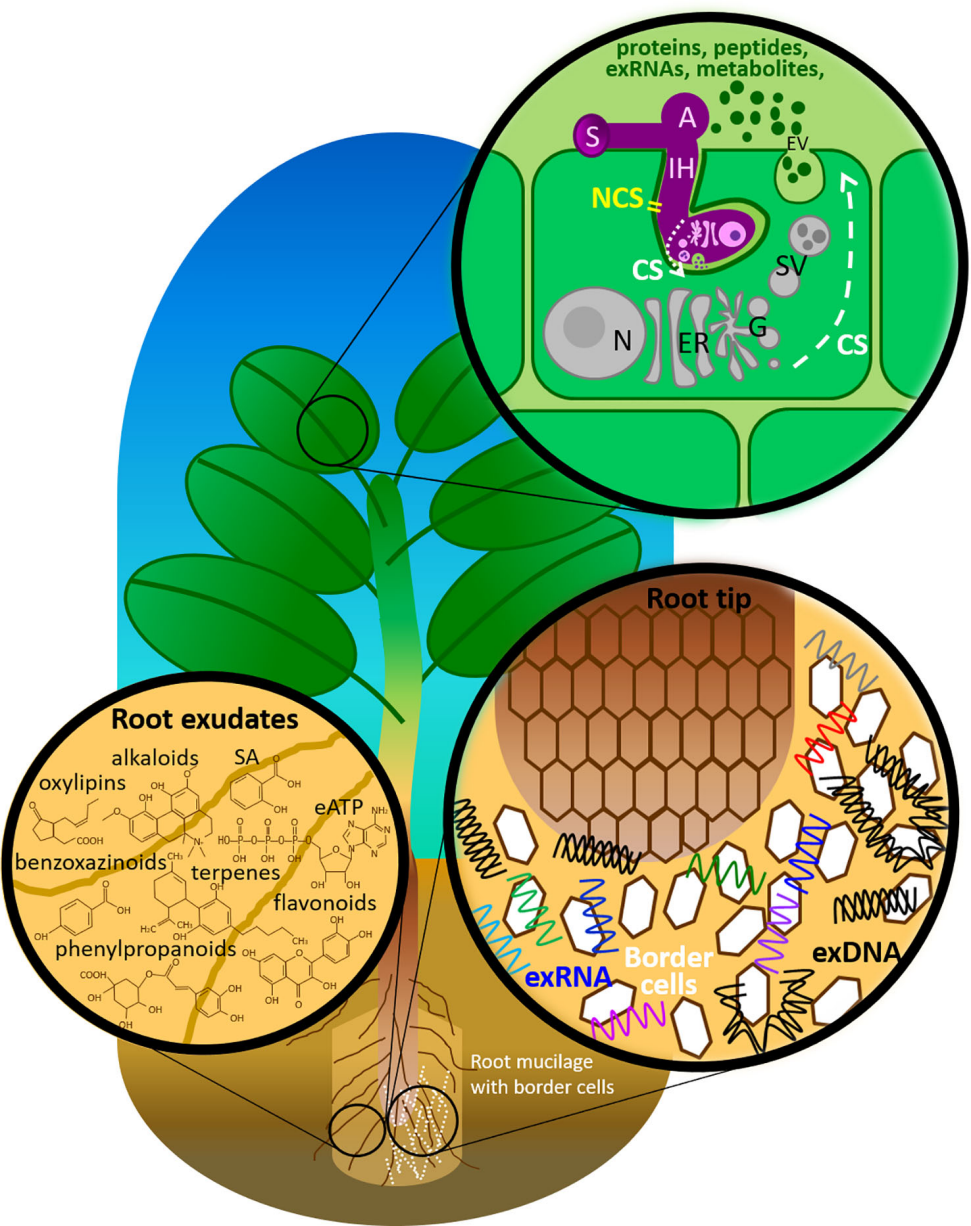
Components of the plant immune system deployed in the extracellular milieu against fungal pathogens. A, appressorium; CS, conventional secretion; eATP, extracellular adenosine 5′-triphosphate; ER, endoplasmic reticulum; EV, extracellular vesicle; G, Golgi apparatus; IH, invasive hyphae; N, nucleus with nucleolus; NCS, non-conventional secretion; SA, salicylic acid; SV, secretary vesicle; S, spore.

Besides proteins, plants and their interacting fungi secrete also a wide variety of molecules, such as peptides, metabolites, phytohormones and nucleic acids (**Figure 1** and **Table 1**). A number of studies have documented the secretion of metabolites by plant roots, but this is often referred to as exudation rather than a secretion process, even though there is compiling evidence that these small molecules can be secreted not only by passive diffusion but also by making use of specific transporters (Baetz and Martinoia, 2014). In addition, EVs are suggested to drive the universal evolution of ribonucleic acid (RNA) export. Microbial RNAs transported in EVs mediate intra- and inter-kingdom communications, including plants’, by regulating gene expression in target cells directly and indirectly *via* host immune receptor signaling (Tsatsaronis et al., 2018).

**Table 1:** List of studies demonstrating the secretion of molecules involved in plant immune response. Grey highlights molecules secreted by fungi.

Plant	Mutant/Plant organ	Partner	Vehicle	Secreted molecule	Targets/modulator	Reference
*Arabidopsis thaliana* (Mouse-ear cress) and *Solanum lycopersicum* (Tomato)	leaf	*Botrytis cinerea*	EV	siRNA effectors	EVs of fungal pathogens in cross-kingdom interaction; fungal plant pathogens siRNA effectors	Weiberg et al., 2013
*Nicotiana benthamiana* (Tjuntiwari and Muntju)	leaf	*Phytophthora infestans*		RXLR effectors	pathogen virulence	Wang et al., 2019
*Zea mays* (Maize)	root apoplast	*Trichoderma virens*		secreted proteins	suppression of plant immune responses	Nogueira-Lopez et al., 2018
	*in vitro* fungal secretome	*Phytophthora infestans*		Leaderless secreted proteins (LSPs), effectors	pathogen virulence	Meijer et al., 2014
	predicted fungal secretome	*Aspergillus fumigatus*		Small secreted and effector-like proteins	pathogen virulence	Vivek-Ananth et al., 2018
*Arabidopsis thaliana*	e*in2* mutant insensitive to eNAD	*Pseudomonas syringae* pv maculicola ES4326	exocytose, transporters	NAD	plant defense	An et al., 2016
*Oryza sativa* (Rice)	*cks1* mutant of *Magnaporthe oryzae*	*Magnaporthe oryzae*		cytokinins	fungal virulence	Chanclud and Morel, 2016
Tomato	leaves; mutants of *Botrytis cinerea* with reduced virulence	*Botrytis cinerea*		botrydial, botcinic acid	secondary metabolites (phytotoxins) in fungal virulence	Dalmais et al., 2011
*Arabidopsis thaliana*	apoplast; *Arabidopsis thaliana* mutants accumulating nucleosides in the extracellular space	*Botrytis cinerea*	transporters	ATP, NAD	susceptibility toward *B. cinerea* infection	Daumann et al., 2015
Rice	suppression of pathogen-induced IAA accumulation	*Magnaporthe oryzae, Xanthomonas oryzae* pv oryzae and *Xanthomonas oryzae* pv oryzicola	transporters	auxin (IAA)	broad-spectrum disease resistance in rice	Fu and Wang, 2011
Rice	*Magnaporthe oryzae* mutants deficient in siderophore biosynthesis	*Magnaporthe oryzae*		siderophore	fungal virulence	Hof et al., 2007
Maize	rhizosphere microbiote	*Spodoptera frugiperda*		secondary metabolites: benzoxazinoids	root exudate, soil microbiote	Hu et al., 2018
Rice	RNAi-knockdown of key regulators of the SA signaling pathway in rice	*Magnaporthe oryzae*		cytokinins	fungal virulence	Jiang et al., 2013
*Arabidopsis thaliana*	rhizosphere microbiome, *Arabidopsis thaliana* mutants in the SA pathway	root microbiote		salicylic acid	root exudate, soil microbiote	Lebeis et al., 2015
Tomato	rhizosphere microbiome	*Trichoderma harzianum*		peroxidases and oxylipins	root exudate, soil microbiote	Lombardi et al., 2018
Maize		*Ustilago maydis*		cytokinins	fungal virulence	Morrison et al., 2017
*Vitis vinifera* L. (Grapevine)	grape berry, apoplast	*Botrytis cinerea*	transporters, extracellular vesicles	flavonoids	defense against phytopathogens; hypersensitive-like response in grapevine leaves	Petrussa et al., 2013
Rice	leaves	*Magnaporthe oryzae*		tyrosine-derived cytochalasan compound	M. oryzae avirulence signalling compound mediated by the PKS-NRPS ACE1	Song et al., 2015
*Arabidopsis thaliana*	roots		exudates	nucleosides, deoxynucleosides, aromatic AAs, anabolites and catabolites of glucosinolates, dipeptides, indolics, SA and JA catabolites, coumarins, mono-, di- and trilignols, hydroxycinnamic acid derivatives and oxylipins		Strehmel et al., 2014)
*Arabidopsis thaliana*	leaves	*Botrytis cinerea*		ATP	induction of plant defense responses	Tripathi et al., 2018
*Medicago truncatula* (Barrel medick)	root border cells			root exudates: 70 compounds identified by metabolomics include AA, OA, sugars, beta-alanine, urea, phenolics, saponins/sapogenins	plant-microbe signaling, defense, and interactions	Watson et al., 2015
*Helianthus annuus* (Sunflower)	extracellular fluid of seedling	*Sclerotinias clerotiorum*	plant EV	proteins	Sunflower pathogenesis-related proteins and defense proteins	Regente et al., 2017
*Arabidopsis thaliana*	apoplast		plant EV	exRNA	*Arabidopsis* EV-transported tiny RNAs (tyRNA)	Baldrich et al., 2019
*Gossypium hirsutum* (Cotton), *Arabidopsis thaliana*	leaf	*Verticillium dahliae*		exRNA	*V. dahliae* genes encoding a Ca2+-dependent cysteine protease (Clp-1) and an isotrichodermin C-15 hydroxylase (HiC-15)	Zhang et al., 2016
*Arabidopsis thaliana*	protoplast	*Botrytis cinerea*	exosomes	exRNA	transferred *Arabidopsis* sRNA TAS1c-siR483, TAS2- siR453	Cai et al., 2018
*Arabidopsis thaliana*	aerial part	*Verticillium dahliae*		exRNA	*Arabidopsis* RNA-silencing components AGO1, AGO7, DCL4, NRPD1a, RDR2, RDR6/SGS2, SDE1, HEN1, HST, SGS3, and SGS1	Ellendorff et al., 2009
*Arabidopsis thaliana*	stem	*Cuscuta campestris*		exRNA	*Arabidopsis* miRNAs encoding the TIR1, AFB2, and AFB3 auxin receptors; the pathogen-induced BIK1 kinase; the injuruy-induced phloem protein SEOR1; and the predicted transcriptional repressor HSFB4	Shahid et al., 2018
*Nicotiana tabacum* (Tobacco)	stem	*Cuscuta pentagona*		exRNA	*C. pentagona SHOOT MERISTEMLESS-like Cp-STM*	Alakonya et al., 2012
*Arabidopsis thaliana*, Tomato	leaf, fruit	*Botrytis cinerea*		*Botrytis cinerea* small RNAs (Bc-siR3.2, Bc-siR3.1, Bc-siR5)	*mitogen activated protein kinase (MPK1, MPK2), Peroxiredoxin-2F (PRXIIF)*, *cell wall-associated kinase (WAK)* from host	Weiberg et al., 2013
Various plants	leaf, fruit, petal	*Botrytis cinerea*		plant exRNA	*B. cinerea* Dicer-like protein 1 (Bc-DCL1) and Bc-DCL2	Wang et al., 2016
*Pisum sativum* (Pea), Tomato	root mucilage		border cell	exDNA	*R solanacearum* extracellular DNases *Rs-nucA, Rs-nucB*	Tran et al., 2016
*Phaseolus vulgaris* (Common bean)	root	itself, *Phaseolus lunatus, Acacia farnesiana*		self *vs.* non-self DNA; exDNA	exDNA as a damage‐associated molecular pattern (DAMP)	Duran-Flores and Heil, 2018
Pea	root tip	*Nectria haematococca*	border cell	exDNA	root tip resistance to fungal infection	Wen et al., 2009
Pea, Maize	root mucilage		border cell	exDNA	root tip defense against pathogens	Wen et al., 2017
*Arabidopsis thaliana*		itself and various microbes		self *vs.* non-self DNA	autotoxicity	Mazzoleni et al., 2015a; Mazzoleni et al., 2015b
Maize	leaf	*Cochliobolus heterostrophus*		exDNase	*C. heterostrophus* TatD type deoxyribonuclease *Ch-NUC1*	Park et al., 2019

The aim of the present review is to summarize the current findings on proteinic and nonproteinic secretion in plants and their interacting fungi, exploring both conventional and nonconventional secretion pathways.

## Secretion of Proteins and Peptides by Plants and Fungi

### Plants: Cargos Are Diverse and So Are Export Processes At the Apoplast Gates

Plants export a wide variety of proteins into the extracellular space, defined in plants as the zone beyond the plasma membrane, i.e. the CW, the apoplast and the rhizosphere. The CW is a dynamic structure constituting a barrier that pathogens need to breach in order to colonize host plant tissues. Plants have developed a sophisticated system for sensing pathogens and monitoring the CW integrity, upon which they activate defense responses that lead to dynamic CW remodeling that prevent disease development. Pathogens, on the other hand, may exploit the host CW metabolism to establish infection. Several studies have documented the role of different classes of secreted proteins in the strategies deployed by both plants and pathogens in this CW battleground. These proteins include CW degrading enzymes (CWDEs), CWDE-inhibitors, CW methylesterases (CWMEs), CWME-inhibitors and oligosacharide-oxidases in the strategies utilized by both plants and pathogens to prevail in this CW battleground. For example, Bellincampi et al. (2014) reported that when pathogens start degrading components of the plant CW, plants are capable of perceiving the loss of CW integrity, and subsequently activate defense signaling pathways. In turn, successful pathogens escape plant defense and take advantage of the host CW metabolism to facilitate their entry into the host tissues. An interesting area of research toward improving plant protection is to study the dynamics of the expression of endogenous and microbial CWDEs and their inhibitors (Bellincampi et al., 2014). Consistent with this, Benedetti et al. (2015) have shown that oligogalacturonides (OGs) released from the degradation of pectin, a major CW component, acted as a damage-associated molecular pattern (DAMP) signal to trigger plant immunity. OGs were generated *in planta* by partial inhibition of pathogen-encoded polygalacturonases (PGs). Under these conditions, plants were more resistant to the phytopathogens *Botrytis cinerea*, *Pectobacterium carotovorum*, and *Pseudomonas syringae*. This strongly supports the idea that controlled release of OGs upon infection may be a valuable tool to protect plants against infectious diseases.

Besides OGs, cellodextrins, which are by-products of cellulose breakdown, are also well-known DAMPs. Recently, Locci et al. (2019) showed enhanced resistance to *B. cinerea* in *Arabidopsis* plants overexpressing a berberine bridge enzyme-like (BBE-like) protein named CELLOX (cellodextrin oxidase), which is the only BBE‐like enzyme identified so far that oxidizes cellulose fragments but not glucose. Restriction of fungal growth might arise from the overexpression of BBE-like enzyme which may have prevented the accumulation of the cellodextrins-type DAMPS. Moreover, oxidized cellodextrins are a less valuable carbon source for the pathogenic fungus, further limiting fungal growth. Based on these results, the authors speculated that other members of the large BBE‐like family may control the homeostasis of CW fragments other than OGs and cellodextrins (Locci et al., 2019). The pectin matrix is the main CW target of *B. cinerea*. Lionetti et al. (2017) showed that pectin methylesterase (PME) activity and pectin methylesterification are dynamically modulated by endogenous PME inhibitors (PMEIs) during the infection, thereby pointing out the role of PMEIs in mediating the maintenance of CW integrity in plant immunity. Interestingly, proteomics analyses of EVs isolated from sunflower seedlings revealed that a large part (112 out of 237 proteins, 47%) of the identified EV proteins are CW-interacting proteins, including enzymes involved in the degradation and reorganization of polysaccharides (de la Canal and Pinedo, 2018). This implicates EVs in the unconventional secretion of CW-modifying enzymes and suggests a role of EVs in modifying the composition of plant CW. Finally, it is worth noting the work of Zhao et al. (2019) showing that yeast strains with deletions in genes involved in CW biosynthesis produce more EVs than the wild type, indicating a potential role for yeast EVs in CW remodeling.

Exported proteins are implicated in a variety of processes other than CW modification, including signaling, development and stress responses (Delaunois et al., 2014). The classical view of secretion of these proteins assumes that they are synthesized and delivered using the conventional ER secretory pathway. Several reviews on this topic have been published (Agrawal et al., 2010; Ghahremani et al., 2016; Martínez-González et al., 2018). A plant protein secreted *via* the canonical secretory pathway presents a N-terminal signal peptide typically 15–30 AAs long, which enables translocation of the protein across the ER in plants and which is cleaved upon secretion (Emanuelsson et al., 2007). The presence of a transit or signal peptide (SP) in secreted proteins can be predicted *in silico* using online algorithms such as signalP or targetP (http://www.cbs.dtu.dk/services) (Nielsen et al., 2019) and the localization of secreted proteins to the apoplast can be *in silico* validated using the online tool apoplastP (http://apoplastp.csiro.au) (Sperschneider et al., 2018b). Proteomics analyses of apoplast fluids have revealed that most pathosystems exhibit conserved biochemical responses involving enzymes acting in CW remodeling (e.g. xylosidases, arabinofuranosidases, fucosidases, pectin methylesterases, galactosidases) as described above, reactive oxidative species (ROS) detoxification (e.g. superoxide dismutases, catalases, peroxidases), as well as pathogenesis-related (PR) proteins (e.g. PR-1, 2, 3, 4, 5, 8, 10, 16 and 17) (Tanveer et al., 2014; Ghahremani et al., 2016; Martínez-González et al., 2018). We refer the reader to these comprehensive reviews for further information on this topic.

### Leaderless Secreted Proteins: The Puzzle of Unconventional Secretion of Proteins

Over the past decade, one of the most intriguing findings in plant secretomics (as well as in fungi secretomics, as discussed below) has been the discovery of a new type of secreted proteins, devoid of a SP, referred to as LSPs (Agrawal et al., 2010; Albenne et al., 2013; Bellucci et al., 2017). Interestingly, LSPs account for more than 50% of the total identified secretome, supporting the existence of novel secretory mechanisms independent of the classical ER–Golgi secretion pathway in plants, which is similar to what has been reported in other eukaryotes. One way these LSPs could be exported to the extracellular milieu is through EVs. In this context, several recent studies led to the characterization of unconventional protein secretory (UPS) pathways that involve EVs for the export of proteins to the extracellular space (Pinedo et al., 2012; Davis et al., 2016; de la Canal and Pinedo, 2018).

Owing to their rigid CWs, the production of EVs in plants had first seemed improbable. We now know that plant cells do indeed secrete EVs, although little is known about the origin, composition and mode of function of EVs (Rutter and Innes, 2018). A protocol for isolating EVs from apoplastic fluids of *Arabidopsis thaliana* leaves, initially devised by Regente and colleagues (Regente et al., 2009) and later implemented with a quantitative fluorometric dye assay to assess total membrane content and screening of the exosome marker PEN1 by Rutter and colleagues (Rutter and Innes, 2017; Rutter et al., 2017), is available. The size of recovered vesicles range between 50 and 300 nm. By using a combination of filtration and differential centrifugation steps, Rutter and Innes (2017) were the first to isolate and purify EVs from *Arabidopsis* leaf apoplasts. The authors also used a proteomics approach to analyze the protein content of purified EVs. EVs are highly enriched in proteins involved in responses to biotic and abiotic stresses, suggesting their likely role in plant defense against pathogens. Consistent with this, EVs secretion was enhanced in plants infected with *P. syringae* and in response to treatment with the plant hormone salicylic acid (SA). Among the recovered defense-related proteins, several proteins involved in signal transmission were identified, many of which were highly induced in response to stress and/or contribute to immunity. Other defense-related proteins included members of the myrosinase–glucosinolate system, involved in ROS signaling, and various membrane-trafficking proteins (e.g. syntaxins, RAB GTPases, patellin family proteins) (Rutter and Innes, 2017). This method was further applied to analyze EVs from sunflower seedlings. In the presence of *Sclerotinia sclerotiorum* ascospores, isolated sunflower EVs were found to be incorporated by fungal cells, which subsequently led to the inhibition of spore germination, stunted mycelial growth, and loss of vitality. Proteomics analyses of EVs taken up by fungal cells have identified several defense proteins (Regente et al., 2017). The biological function of EVs in plant–pathogen interactions is just emerging (Boevink, 2017; Hansen and Nielsen, 2017). Their role as key mediators of such interactions needs to be further investigated.

In parallel to experimental studies, new bioinformatics tools are currently being developed for *in silico* characterization of LSPs. For example, ApoplastP can predict whether an effector candidate or a plant protein localizes to the apoplast and, in doing so, allows the identification of sequence features, common to both effectors and plant proteins, that are required for apoplastic localization. Such sequence properties include depletion in glutamic acid, acidic and charged amino acids as well as enrichment in small amino acids (AAs) (Sperschneider et al., 2018a). SecretomeP is an online tool that provides *ab initio* predictions of LSPs (Bendtsen et al., 2004). Because this algorithm has initially been designed for mammalian proteins it might prove unreliable for plant LSPs (Delaunois et al., 2014). To overcome this shortcoming, a plant secretome knowledgebase PlantSecKB has been developed (https://omictools.com/plantseckb-tool) (Lum et al., 2013). Besides proteins, plant cells synthesize and export an array of peptides originated from larger precursors (thus not different from protein export discussed above) or directly made from small Open Reading Frames (ORFs). For instance, antimicrobial peptides (AMPs) are peptides of up to 100 AAs that are structurally and biochemically highly diverse, are of ribosomal or non-ribosomal origin, and display activities against microbial pathogens. Plant AMPs have various roles, covering functions such as hormones like systemin and HypSys, defense signaling like Pep1 and GmPep914 and GmPep890, or DAMP elicitor like CAPE1, as well as defensins that are constitutively expressed *in planta* and carry antifungal activity [reviewed in (Breen et al., 2015)].

An intriguing question is whether LSPs exported *via* UPS are glycosylated. While it is rational to assume that glycosylation is the exclusive signature of the passage through the ER secretory pathway, very little is known about the subcellular localization of *O*‐ and *N*‐glycosylated enzymes in plants. A study by Poulsen et al. (2014) has presented data demonstrating that at least some of the *O*-glycosyltransferases are localized to unique subcellular compartments that are distinct from Golgi apparatus and that may be part of UPS pathway. Therefore, these data support the view that it is possible for glycosylated proteins to be exported through unconventional pathways. On the other hand, Stock et al. (2016) could show, through heterologous expression of a modified β-glucuronidase (GUS) reported gene in *Ustilago maydis*, that glycosylation only took place when the ER secretory pathway directed protein secretion. No glycosylation was observed in the UPS pathway. This was concluded based on monitoring the activity of secreted GUS enzyme, which is hampered when the protein is glycosylated. GUS activity could not be detected in the supernatant of *U. maydis* culture when GUS was fused to N-terminal SP. Instead, GUS activity was restored when GUS was fused to Chitinase Cts1, an enzyme known to be released *via* UPS in *U. maydis*.

Although it is possible that UPS pathways may be dissimilar in plants and fungi, the above-mentioned puzzling results do not allow to draw clear-cut conclusions regarding whether or not glycosylated proteins are being exported through the UPS pathway. In addition, it is as yet completely unknown to science how many UPS pathways exist in plants and fungi. Indeed, more empirical work is needed to address these questions.

Posttranslational modifications (PTMs) are likely to play an important role, including additional functions associated with protein localization and delivery, incorporation of other cargo proteins/RNAs, or exclusion from secretion (Claridge et al., 2019). More generally, the mechanisms that define the sorting of proteins into EVs and subsequent trafficking are the focus of recent work. Several other basic features regarding EVs’ biology, diversity and function are, to date, simply assumed or based on untested hypotheses that need to be experimentally tested for the field to advance (Margolis and Sadovsky, 2019).

### In Fungi Size Matters: Plant Pathogens Possess the Largest Secretomes

Fungi export large amount of proteins and small peptides, including effectors, into the extracellular milieu. Accumulating genome sequence data has helped understand both the size and nature of fungal secretomes. Pathogenic fungi secrete larger numbers of proteins than symbionts. Among plant and animal pathogens, the largest secretomes are found in crop-infecting necrotrophs, while the smallest secretomes are released by biotrophs. Hemibiotrophs and wood-decaying necrotrophs produce secretomes with intermediate sizes. The small size of biotrophic secretomes is mostly attributed to the fact that this group of fungi lack many proteins with enzymatic activity (Kim et al., 2016).

A recent genome-wide survey of 250 fungal secretomes unveiled the presence of putative KEX2-processed repeat proteins (KEPs) in nearly all fungi, including mycorrhizal fungi and human and plant pathogens. This class of secreted peptides are processed in the Golgi apparatus upon their passage through the secretory pathway and are exported to the extracellular space by exocytosis (Le Marquer et al., 2019a; Le Marquer et al., 2019b). While some KEPs are predicted to be ɑ-type sexual pheromones that play a role in mating, others are predicted to act as mycotoxins and many other KEPs are of unknown function.

Many secreted fungal proteins with enzymatic activities have predicted biological functions, including the breakdown of the host CW, self-protection or nutrient acquisition, such as carbohydrate-active enzymes (CAZymes (Cantarel et al., 2009)), oxidoreductases, proteases, and lipases (Kim et al., 2016; Franceschetti et al., 2017). A net reduction in these enzymes in mycorrhizal fungal genomes has placed these fungi in a separate group (Tisserant et al., 2013; Pellegrin et al., 2015; Garcia and Ane, 2016). However, a large group of secreted putative fungal effectors carry no recognizable Pfam domain (Rafiqi et al., 2012; Vivek-Ananth et al., 2018). This group of unknown secreted proteins is thought to play a crucial role in enabling fungal colonization of plant tissue. Yet, this is the least characterized group of secreted proteins. While the exact biological function in the host is yet to be discovered for most effectors, knowledge is being accumulated on how their expression is regulated upon fungal invasion of plant tissue along with their final host subcellular destination. An interesting study by Zeng et al. (2018) has shown that while some secreted proteins conserve the same expression upon interaction of the arbuscular mycorrhizal fungus *Rhizophagus irregularis* with different plant species, a subset of these proteins are host-specific. A recent study has shown that long-distance retrograde motility of early endosomes is required for the expression of effector genes in *U. maydis* during host invasion (Bielska et al., 2018). A mitogen-activated kinase, Crk1, transported in early endosomes is involved in the retrograde signaling that coordinates effectors expression. Wang and colleagues have used fusion to fluorescent proteins and heterologous expression in the model host *Nicotiana benthamiana* to identify the subcellular targets of 52 *Phytophthora infestans* effectors (Wang et al., 2019). While most of these effectors are nucleocytoplasmic, many others target more specific localizations, including the plasma membrane, the ER, microtubules or host organelles such as the nucleus, indicating the vast diversity of host subcellular compartments targeted by the pathogen and, consequently, indicating where host molecular interactors might be located. The diversity of the final cellular targets of fungal effectors strengthens the hypothesis that adapted coevolved pathogens use far-reaching and diverse strategies that, when combined, have the potential to take control over molecular processes in infected host cells in favor of disease development.

### At the Frontline: Plant and Fungal Secretomes Merge

Plants recognize molecules of pathogens or microbes that attempt infection. These molecules are referred to as invasion patterns (IPs) or invasion molecules (IMs) (Cook et al., 2015; Kanyuka and Rudd, 2019) and were previously known as microbe- or pathogen-associated molecular patterns (MAMPs or PAMPs) in the original Zigzag model proposed by Jones and Dangl (2006). Despite the importance of this early immune response, relatively little is known about the size and nature of the plant secretomes deployed to control microbial and pathogenic invasions. Upon perception of IMs in the apoplastic space, plants secrete an arsenal of proteins and peptides with the primary function of halting fungal growth (Breen et al., 2015; Gupta et al., 2015). When secreted plant proteins encounter the fungal secretome, intensive biochemical interactions are expected to take place at the fungal–plant interface. It is expected that all microbes must face this first layer of the plant immune system.

Necrotrophic pathogens, which kill the cells they infect, secrete a range of proteins with enzymatic activity (discussed above) or protein effectors that exploit host cell death mechanisms to promote pathogen growth and cause disease. One example is SnToxA effector from wheat-infecting *Stagonospora nodorum* (Vincent et al., 2012) In contrast, biotrophic and mutualistic fungi, which grow and reproduce on living plant cells, deploy more exquisite secretomes. *Trichoderma virens* is a biocontrol and opportunistic endophytic fungus (Harman et al., 2004). A recent study on the interaction of *T. virens* with maize roots (Nogueira-Lopez et al., 2018) has identified 95 secretory proteins of maize using a gel-free shotgun proteomics approach. In this study, maize secretome was found to be reduced by 36% upon colonization with *T. virens* (Nogueira-Lopez et al., 2018). Seven secreted Uaca_Ns effectors from the bean rust pathogen *Uromyces appendiculatus* were shown to suppress plant host innate immunity, by either dampening pathgogen-triggered immunity or preventing hypersensitive response (Qi et al., 2019).

Such proteomics analyses will be further needed when comparing secretomes of susceptible *versus* resistant plants, as it will help identify critical components that underlie resistance *versus* susceptibility in the host, and virulence *versus* avirulence in the pathogen. This in turn will help develop pathogen control strategies through enhancing resistance traits in the host (e.g. *via* introgression of resistance genes into commercial varieties or *via* GMO approach) and through suppression of virulence genes in the pathogen (e.g. *via* agrochemical development or *via* RNAi mechanisms discussed below).

### Are We Missing Something? Non-Conventional Secretory Pathways in Fungi

Proteinic secretomes of fungi have so far been identified through mining fungal genomes using bioinformatics pipelines (Saunders et al., 2012) that filter out proteins harboring SP and consequently being predicted to be exported outside fungal cells through the classical ER secretory pathway. However, an increasing number of experimental studies recover proteins lacking SP within fungal and oomycetes secretomes. For example, Meijer and colleagues have recovered, through mass spectrometry, proteins lacking obvious SP when analyzing proteins exported by *P. infestans* in different growth media (Meijer et al., 2014). Similarly, Nogueira-Lopez and colleagues have found evidence for non-conventional secretion mechanisms when analyzing the secretomes of *T. virens* on its own and within infected maize cells (Nogueira-Lopez et al., 2018).

A recent analysis of the secretome of *Aspergillus fumigatus* and ten other related species has also concluded that 64 A*. fumigatus* proteins (0.65% of the proteome) are secreted through unconventional mechanisms, as opposed to 598 (6.1% of the proteome) that are secreted through the ER secretory pathway (Vivek-Ananth et al., 2018). Altogether, these findings point to the necessity of considering experimental approaches to characterize secretomes combined with *in silico* methods in order to ensure a comprehensive analysis of plant and fungal secretomes.

Several methods are now available for the identification of secreted proteins both in growth media as well as in the apoplastic space (Tanveer et al., 2014; Gupta et al., 2015; Miura and Ueda, 2018). Inclusion of empirical analyses, in addition to bioinformatics prediction, when identifying fungal extracellular proteins is of high importance. Empirical approaches are increasingly needed to identify the whole spectrum of extracellular proteins that play a role not only in fungal colonization of plant tissue, but also those deployed in fungal cell growth and modifications.

## Beyond Proteins: What Else Do Plants and Fungi Secrete?

Secretome studies in plants and their interacting fungi most often address the role of proteins, including effectors that are secreted in the extracellular space (Kamoun, 2006; Agrawal et al., 2010; Ghahremani et al., 2016; Cologna et al., 2018). However, both plants and fungi secrete molecules other than proteins, notably small molecules such as metabolites, secondary metabolites and hormones (**Figure 1** and **Table 1**). In the following sections, we review the secretion of such nonproteinic molecules by plants and their interacting fungi.

### Export of Metabolites by Plants

#### Root Exudates

Root exudation is an important source of organic carbon in the soil, which accounts for up to 2–10% of the total photosynthetic production (Kuzyakov and Domanski, 2000). This leads to a process called “soil priming”, whereby the microbial community becomes more active and liberates nutrients that are important for plants (Fontaine et al., 2013; Gargallo-Garriga et al., 2018). Plant roots release complex mixtures of bioactive molecules, including those affecting the activity and composition of the rhizosphere microbiota (Sasse et al., 2018). For example, tomato root exudates, which act as chemoattractants of the biocontrol fungus *Trichoderma harzianum*, were shown to contain peroxidases and oxylipins, both known to be released by roots in response to stress (Lombardi et al., 2018). Other examples are benzoxazinoids, a class of defensive secondary metabolites released by roots of cereals such as wheat and maize. Benzoxazinoids alter fungal and bacterial communities associated with roots and increase plant defenses (Hu et al., 2018). Similarly, studies conducted on different plant-metabolites concluded that secondary metabolites contained in root exudates, including alkaloids, flavonoids and phenolics, can potentially combat bacterial, viral and fungal infections (reviewed by (Zaynab et al., 2018)). Unexpectedly, SA, a major phytohormone usually involved in plant defense in the shoot system, proved also able to sculpt the microbiota of *Arabidopsis* roots (Lebeis et al., 2015).

The chemical composition of root exudates was explored through metabolomics studies (Strehmel et al., 2014; Watson et al., 2015; van Dam and Bouwmeester, 2016; Gargallo-Garriga et al., 2018), notably through the recent field of exometabolomics that aims at understanding how such exudates inhibit pathogen growth or, on the contrary, recruit mutualistic microbial species (Jacoby and Kopriva, 2018; Zhalnina et al., 2018). Metabolomics revealed the large chemical diversity of root exudates. Consequently, a number of compounds including nucleosides, deoxynucleosides, aromatic AAs, anabolites and catabolites of glucosinolates, dipeptides, indolics, SA and jasmonic acid (JA) catabolites, coumarins, mono-, di- and trilignols, hydroxycinnamic acid derivatives and oxylipins, were identified in *Arabidopsis* root exudates (Strehmel et al., 2014) and *Medicago truncatula* root border cells (Watson et al., 2015).

Vesicles are involved in secondary metabolites and flavonoids transport in plant cells (Petrussa et al., 2013). Membrane-bound proteins are implicated in metabolite release from plant cells. Some localize to the plasma membrane where they can directly export compounds from the cell. Others are associated with internal membranes where they may sequester compounds within subcellular compartments or perhaps load vesicles ready for exocytosis. These proteins include the ATP-binding cassette (ABC) family, the Multidrug and Toxic Compound Extrusion (MATE) family, the Major Facilitator Superfamily (MFS), and the Aluminum-Activated Malate Transporter (ALMT) family of transport proteins (Badri et al., 2008; Badri et al., 2009; Badri and Vivanco, 2009; Weston et al., 2012; Ziegler et al., 2017). Therefore, both conventional (transporters) and unconventional (extracellular trafficking) pathways ensure the secretion of plant secondary metabolites.

Similarly, fungal pathogens release volatile substances, such as the sesquiterpene-derived trichotecene toxins from *Fusarium culmorum* that are potent inhibitors of protein synthesis and inhibit the activation of plant-defense response genes prior to any physical contact with the pathogen (reviewed in (Zeilinger et al., 2016)). Transporters are involved in the secretion from fungal cells of several secondary metabolites and phytohormones (Yazaki, 2006; Badri and Vivanco, 2009; Lefevre and Boutry, 2018).

#### Extracellular Nucleosides and Nucleotides

One secreted metabolite, adenosine 5′-triphosphate (ATP), was the object of several studies in plants. While traditional studies emphasized the roles of nucleotides in intracellular energy metabolism, recent findings, first in animals then in plants, highlighted their potential roles outside the plasma membrane. Extracellular ATP (eATP) plays critical roles in plant stress responses, suggesting that it can act as a DAMP. A major finding arose from the characterization of the ATP-insensitive *Arabidopsis* mutant, *dorn1* (*Does not Respond to Nucleotides 1*), which is defective in the lectin receptor kinase I.9. The data disclosed that DORN1 protein binds eATP with high affinity and is required for ATP-induced calcium response, mitogen-activated protein kinase (MAPK) activation, and gene expression. Thus, DORN1 is essential for the perception of eATP and as such plays a variety of roles in plant stress resistance. DORN1 is the founding member of a new plant-specific purinoceptor subfamily, P2K (P2 receptor kinase). It consists of an extracellular legume L-type lectin domain, a transmembrane domain, and an intracellular serine/threonine kinase domain. The predicted structure of the extracellular domain revealed putative key ATP binding residues (Choi et al., 2014a; Choi et al., 2014b; Tanaka et al., 2014; Cho et al., 2017). Another study on the susceptibility to *B. cinerea* infection in *Arabidopsis* mutants accumulating nucleosides in the extracellular space was addressed. These plants markedly accumulated adenosine and uridine in leaves, were highly susceptible toward *B. cinerea* and showed a reduced induction of pathogen-related genes *PR1* and *WRKY33* (Daumann et al., 2015; Feng et al., 2015).

In addition, eATP is also implicated in plant-fungi symbiotic interactions. For example, the beneficial root endophyte *Serendipita indica* secretes SiE5′NT, an enzymatically active ecto‐5′‐nucleotidase capable of hydrolyzing nucleotides in the apoplast. Importantly, *Arabidopsis* lines producing extracellular SiE5′NT were significantly more colonized, had reduced eATP levels, and altered responses to biotic stress, indicating that SiE5′NT functions as a compatibility factor (Nizam et al., 2019).

As in animals, ATP appears to be released by plant cells *via* vesicular exocytosis (Marzec, 2011; Tanaka et al., 2014), thereby highlighting the involvement of unconventional secretion mechanisms. However, in *Arabidopsis*, AtPGP1, an ABC transporter, and PM-ANT1, a plasma membrane-localized nucleotide transporter, were shown to export intracellular ATP into the extracellular milieu by conventional secretion (Tanaka et al., 2014). Therefore, the secretion of eATP by plant cells makes use of both unconventional and conventional mechanisms. No information is presently available on the existence of eATP in fungi.

#### Phytohormones

Several recent studies pointed out the central role of phytohormones, including SA, JA, auxins, cytokinins (CKs), abscisic acid (ABA), in regulating plant-microbe interactions (Lebeis et al., 2015; Boivin et al., 2016; Di et al., 2016; Carvalhais et al., 2017). Of importance is the discovery that SA sculpts the *Arabidopsis* root microbiome. Thus, isogenic *Arabidopsis* mutants lacking biosynthesis of SA, and/or SA-related signaling, display root microbiota that differ in the relative abundance of specific bacterial families relative to those of wild type (Lebeis et al., 2015). This study demonstrated that different bacterial strains could make use of SA in different ways, whether as a growth signal or as a carbon source. Together, these findings show that a central regulator of the plant immune system, largely uncharacterized in roots, directly influences the root microbiota composition (Lebeis et al., 2015). Consistent with this, exogenous SA and derivatives proved able to inhibit the growth of *B. cinerea in vitro* (Dieryckx et al., 2015). Similarly, several studies showed that ABA exhibits antifungal activity in plants (Khedr et al., 2018).

### Export of Small Molecules by Phytopathogenic Fungi

#### Phytohormone-Mimicking Compounds

It is established that classical plant hormones, including auxins, CKs, gibberellins (GAs), ABA, ethylene, SA, JA, or metabolites mimicking phytohormones, are produced by plant-interacting fungi (Robert-Seilaniantz et al., 2007; Chanclud and Morel, 2016). Converging evidence suggests that these fungal-derived molecules can perturb plant processes, either positively or negatively, notably to favor fungal invasion (Chanclud and Morel, 2016; Ma and Ma, 2016; Vrabka et al., 2018; Zhang et al., 2018). For example, upon infection of rice by the blast fungus *Magnaporthe oryzae*, diverse CK species could be detected in the hyphae, conidia, and culture filtrates, indicating that the fungus is capable of producing and secreting CKs (Jiang et al., 2013). In agreement with the role of CKs in plant infection by fungal pathogens, CK-deletion strains of the smut fungus *U. maydis* elicited fewer and smaller tumors than the pathogenic strain SG200. Consistent with this, mining of the *U. maydis* genome identified genes encoding CK signaling- and biosynthesis-related proteins (Morrison et al., 2017). Also, diverse pathogens are able of synthesizing and secreting indole-3-acetic acid (IAA), the major form of auxin in plants. This secretion of IAA may have a direct virulence effect by loosening the plant CW, opening stomata, and inhibiting SA-dependent defense signaling (Fu and Wang, 2011).

Furthermore, several phytopathogenic fungi, such as *Cercospora rosicola*, *B. cinerea* and *M. oryzae* have the ability to produce ABA through a biosynthetic pathway that is distinct from that of plants (Izquierdo-Bueno et al., 2018). Impairing ABA biosynthesis in *M. oryzae* dramatically reduces virulence (Spence et al., 2015).

The endophytic fungus *A. niger* CSR3 produces IAA and GAs to promote growth of the GA-deficient rice mutant Waito-C (Lubna Asaf et al., 2018). Multifunctional plant growth-promoting fungi (PGPF) include the fungal genera *Aspergillus, Fusarium, Penicillium, Piriformospora, Phoma*, and *Trichoderma*. The associations between plants and multipurpose PGPF have been shown to control numerous foliar and root pathogens through induced systemic resistance (ISR) in host plants, notably through phytohormones production (Hossain et al., 2017).

#### Phytotoxic Fungal Metabolites

Necrotrophic fungi secrete phytotoxic secondary metabolites to kill the host tissues and suppress plant-defense responses (Osbourn, 2001; Keller et al., 2005; Muria-Gonzalez et al., 2015; Pusztahelyi et al., 2015; Collemare et al., 2019). These metabolites are secreted into infected plants and either act as virulence factors, i.e. intensify disease symptoms, or act as pathogenicity factors, i.e. are exclusively responsible for the development of disease symptoms. *B. cinerea* secretes several toxic compounds, including the secondary metabolites botrydial (a sesquiterpene) and botcinic acid (a polyketide), causing the death of infected plant cells (Dalmais et al., 2011). Other examples of secondary metabolites essential for fungal virulence are siderophores. *M. oryzae* produces the siderophore ferricrocin, which contributes to pathogenicity on rice by interfering with appressorium turgor pressure (Hof et al., 2007).

Of interest is the finding that *M. oryzae ACE1*, which encodes a polyketide synthase-nonribosomal peptide synthetase (PKS-NRPS) fusion protein, differs from other fungal avirulence (AVR) genes in that it is not a secreted protein, despite behaving like a classical AVR gene. When carrying functional AVR gene *ACE1*, *M. oryzae* is unable to infect rice cultivars carrying the corresponding *Pi33* resistance gene, whereas isolates or mutants defective for *ACE1* are virulent and bypass the rice Pi33-mediated resistance. ACE1 accumulates exclusively in appressoria during fungal penetration of host tissue. Mutation of its β-ketoacyl synthase domain abolishes recognition of the fungus by resistant rice, indicating that ACE1 enzyme activity is required for avirulence. These findings provide evidence that the avirulence signal recognized by Pi33 rice cultivars is not the ACE1 protein itself, but rather the secondary metabolite (most presumably a tyrosine-derived cytochalasan compound) synthesised by this enzyme and exported into infected plant cells (Collemare et al., 2008a; Collemare et al., 2008b; Song et al., 2015).

#### Export Mechanisms of Fungal Small Compounds

In animal systems, it is well established that vesicular trafficking plays a key role in fungal secondary metabolism and transport. For example, this has been documented for the filamentous fungus *A. parasiticus* in relation with the transport of aflatoxin, a secondary metabolite considered as one of the most potent naturally occurring carcinogens known to date (Chanda et al., 2009). These studies allowed the discovery of aflatoxisomes, which are specialised trafficking vesicles that are implicated in the export of the toxin outside the cell (Roze et al., 2011). Moreover, endosomal trafficking was shown to be critical for subcellular localization of melanin biosynthetic enzymes in the human fungal pathogen *A. fumigatus* (Upadhyay et al., 2016).

Information on extracellular vesicles in the transport of secondary metabolites from plant pathogenic fungi remains scarce. However, endosomes were suggested to be implicated in the synthesis and secretion of aflatoxins by *A. parasiticus* (Chanda et al., 2010). Thus, this vesicular‐mediated secretion could provide a route for fungal export of metabolites that is distinct from efflux by membrane transporters. It is anticipated that vesicular-mediated secretion in plant pathogenic fungi will become an active topic of research in the near future (Collemare et al., 2019). Conventional export/secretion of secondary metabolites seems to operate in plant pathogenic fungi as they possess several ABC and MFS transporters (Amnuaykanjanasin and Daub, 2009; Coleman and Mylonakis, 2009).

## The New Nucleic Acid Kids on the Block: Secretion of Rna and Dna by Plants and Fungal Phytopathogens

In the previous sections, we highlighted how plant-fungal interactions are mediated by the exchange of diverse proteins, peptides and metabolites between both partners. Here, we address the export of nucleic acids (RNA and deoxyribonucleic acid (DNA)) as novel secreted molecules (**Figure 1** and **Table 1**).

### Extracellular RNA

Extracellular RNA (exRNA) comprises RNA molecules located outside cells through active secretion mediated by EVs or membrane transporters, or through passive release from apoptotic cells (Tsatsaronis et al., 2018). ExRNAs include rRNAs, tRNAs, mRNAs, and small RNAs (sRNAs). sRNAs are short [21- to 24-nucleotides (nt)] non-coding regulatory RNAs resulting from cleavage of double-stranded RNA substrates by dicer-like (DCL) enzymes. Small RNAs silence genes whose sequences are complementary. Small RNAs are further subcategorised into microRNAs (miRNAs) and small interfering RNAs (siRNAs), based on the differences in their biogenesis and modes of action. They move between hosts and interacting organisms and induce gene silencing, known as cross-kingdom/organism RNA interference (RNAi) (Cai et al., 2018). sRNAs are transferred in a selective fashion across cells and tissues of individuals and across species, thus connecting animal, plant, and microbial kingdoms.

Such is the interest in this active field of research that the Extracellular RNA Communication Consortium (ERCC) was launched in 2013 (Ainsztein et al., 2015) with its first progress report released this year (Das et al., 2019). Aware of the role of exRNAs in the intercellular communications in human, the ERCC aimed at profiling exRNAs and their carriers from diverse biofluids from healthy individuals, identifying exRNA biomarkers for a broad range of diseases and optimizing methods for large-scale production of safe and effective exRNA- and EV-based therapeutics. Significant progress was achieved through refined sample preparation and next generation sequencing (NGS) methods. This has enabled the exploration of the entire RNA populations from various samples and treatments over a time-course. However, mining this large amount of data remains a challenge (Morgado and Johannes, 2017).

Compared to human sRNAs, research on plant and fungal sRNAs and EVs has been lagging behind. However, it is quickly catching up as illustrated by the number of reviews published on this topic (Weiberg et al., 2014; Han and Luan, 2015; Bhat and Ryu, 2016; Islam et al., 2017; Hua et al., 2018; Rutter and Innes, 2018; Tsatsaronis et al., 2018; Zhao et al., 2018; de Vries et al., 2019; Lee, 2019; Zeng et al., 2019).

#### Plant Extracellular sRNAs and Vesicles

In plants, sRNAs have key regulatory functions in development, physiology, response to biotic and abiotic stresses, genome stability and transposon control (Mirouze, 2012). Plants transport viral RNAs, mRNAs, miRNAs and siRNAs through the phloem (Kehr and Buhtz, 2008). Loading of RNA into the phloem is likely to be facilitated by plasmodesmata and associated RNA-binding proteins. EVs represent an alternative process for the export of RNAs into the phloem and possibly the transport of RNA through the phloem or apoplast (Rutter and Innes, 2018).

EVs were isolated from apoplastic fluids of *Arabidopsis* leaves (Baldrich et al., 2019) and analysed for sRNAs. *Arabidopsis* EVs contain a high number of sRNAs whose sises, classes and identities vary, including miRNAs, siRNAs, as well as so-called tinyRNAs (tyRNAs, 10–17 nt-long) of unknown function and originating from mRNA, rRNA and miRNA precursors. Conversely, apoplastic fluids devoid of EVs are enriched in sRNAs able of inducing silent expression in genes with matching sequences, which led to hypothesise on the existence of two independent pathways for sRNA export, with or without EVs as vehicles (Baldrich et al., 2019). Analysis of whole tissue sRNA public datasets in repositories revealed the over-representation of tyRNAs in plants compared to mammals. However, tyRNAs have been overlooked due to bioinformatic analysis pipelines that discard reads shorter than 18 nt. Adjusting pipelines parameters to include such tyRNAs should help find out whether plant tyRNAs are taken up by pathogens *via* uptake of EVs, and will help reveal their potential role in plant immunity (Bielska et al., 2019).

#### Extracellular sRNAs Associated With Plant Immunity Against Fungi

The involvement of extracellular miRNA in the plant immune system has become apparent in the seminal study of cotton plants infected by *Verticillium dahliae* (Zhang et al., 2016). Cotton plants increased expression of native cotton miRNAs Gh-miRNA166 and Gh-miR159 in response to *V. dahlia* infection. Both miRNAs were delivered into the fungus, *via* as yet an unknown process, where they targeted genes essential for pathogen virulence, namely a Ca^2+^ dependent cysteine protease (Clp-1) and an isotrichodermin C-15 hydroxylase (HiC-15) (Zhang et al., 2016). Future research should investigate the mechanisms of miRNA export from the host plant into fungal pathogens and translational applications.

A recent outstanding study of the *Arabidopsis–B. cinerea* pathosystem by Cai and colleagues (2018) developed a sequential protoplast purification method to isolate pure fungal cells from infected plant tissues. A number of *Arabidopsis* siRNAs were identified inside *B. cinerea* cells that would have been transferred not merely through concentration-dependent diffusion but possibly through a more selective process mediated by EVs. Conducting such comprehensive analyses in other pathosystems will highlight how prevalent this immune strategy is in the plant kingdom.

The combined facts that sRNAs contribute to plant immunity, transit through and between organisms, and act rapidly on multiple targets conserved across different species make them promising candidates to be considered in disease resistance breeding programs for valuable crops (Rose et al., 2019). The method of Host-Induced Gene Silencing (HIGS) exploits the silencing effect of sRNA signals in interacting organisms and involves host expression of sRNA-generating constructs directed against genes in associated pathogens to dampen their virulence. The efficacy and persistence of disease resistance reported in tightly-controlled laboratory conditions remains to be validated under field conditions (Knip et al., 2014).

#### Family Feud: Plant Virulence Against Other Plant Species

Plant crops fall not only victim to the molecular clutches of various microbial pathogens but also to those of weeds and parasitic plants. Connection with the host can take place by root invasion, as with *Striga hermonthica*, or by constricting and invading the host’s aerial photosynthetic parts and stem, as with *Cuscuta* (dodder) genus (Johnson and Axtell, 2019). Through physical connections between *Cuscuta* and its hosts, movements of water, nutrients and metabolites take place, carrying along macromolecules such as proteins and mRNAs (Kim and Westwood, 2015; Westwood and Kim, 2017). Due to its relative wide host range, *Cuscuta* can parasitize several species from diverse range of plant families, and therefore act as a sink for host mobile RNA from many different species. Furthermore, host mRNA sequences are highly divergent from those of *Cuscuta*, thus simplifying the bioinformatical process of filtering out host mRNA that has trafficked into the parasite (Leblanc et al., 2012). Consequently, an impressive body of knowledge has recently been accumulated on RNA trafficking in this system (for review, see afore-mentioned references).

Naturally occurring sRNAs can be exchanged across the parasite haustorium thereby affecting gene expression in the host plant. *C. campestris* haustoria accumulate high levels of many novel miRNAs, 22-nt long, while parasitizing *Arabidopsis* plants. miRNAs of 22-nt occur less frequently than 21-nt miRNAs in plants and are often associated with accumulation of secondary siRNA from their targets. *C. campestris*-derived miRNAs are active inside host cells and hijack the host’s own silencing machinery to produce secondary siRNAs. Together, these data suggest that *C. campestris* trans-species miRNAs function as virulence factors to remodel host gene expression to the parasite’s advantage (Shahid et al., 2018).

*Cuscuta* is susceptible to HIGS, as exemplified hereafter. Interspecific sRNA-mediated silencing of parasite genes is one of the strategies to produce crops resistant to dodder invasion. A likely candidate would be the KNOTTED-like homeobox transcription factors, including SHOOT MERISTEMLESS-like (STM), which is essential to the development and subsequent establishment of haustorial connections by *C. pentagona* on tobacco plants. Interspecific silencing of the STM gene in dodder driven by a vascular-specific promoter in transgenic host plants disrupted dodder growth (Alakonya et al., 2012). HIGS technology is therefore a potential effective method for control of infection by plant parasites.

### Extracellular DNA

Intracellular or internal DNA (iDNA) is the DNA located within the cell membranes while extracellular, external or environmental DNA (exDNA) represents the DNA located outside cells originating from iDNA by active or passive extrusion mechanisms and/or by cell lysis (Pietramellara et al., 2009). A method to discriminate between iDNA and bound or free exDNA, as well as evaluate various DNA fractions and infer related ratios (ex:iDNA) was developed for microbes, including anaerobic fungi (Nagler et al., 2018b). A thorough overview on the main research areas dealing with exDNA, its origins and functions, and existing and emerging exDNA-based methods and applications was recently published (Nagler et al., 2018a).

#### Self *Versus* Non-Self DNA

Across the tree of life, from plants to mammals, immune and nonimmune cells express evolutionarily conserved pattern recognition receptors (PRRs) that sense and recognize DNA as a potential marker of self-damage and/or non-self-organisms, which in turn trigger responses to inflammation, immunity, or pathogen resistance. Self-DNA is an indicator of self-damage and acts as a DAMP. Non-self DNA indicate the presence of a foreign organism and act as a PAMP or a MAMP (Gallucci and Maffei, 2017).

As previously explained, we limit the scope of our review to plants and their interacting fungal partners. However, we acknowledge the breakthrough study by Tran and colleagues (Tran et al., 2016) on the tomato bacterial pathogen *Ralstonia solanacearum*. Deletion of two exDNase genes, *nucA* and *nucB*, trapped bacterial mutants in the exDNA matrix of tomato root border cells, resulting in dampening of the pathogen’s virulence. These findings demonstrate for the first time that exDNases are virulence factors deployed by plant pathogens in the counter defense strategy against host exDNA.

Plants not only depend on small molecules or ions to garner mineral nutrients for growth but are also able to absorb into their roots large organic molecules such as proteins used as an alternative source of nitrogen (Paungfoo-Lonhienne et al., 2008) and DNA as an alternative source of inorganic phosphorous (Paungfoo-Lonhienne et al., 2010a). Uptake of non-self exDNA by plant roots was also shown to have a role in signaling by supplementing hydroponic cultures of *Arabidopsis* plants with foreign DNA from herring sperm. The addition of foreign DNA induced changes in expression of *CLAVATA3/ESR* (CLE)-related gene, encoding a peptide hormone implicated in root morphogenesis, that can potentially enhance nutrient absorption by the plant, thus pointing to a DNA-elicited signal pathway (Paungfoo-Lonhienne et al., 2010b).

#### Once Upon a Slime: Root Border Cells Trap Pathogens

Soil-borne plant pathogens invade their plant hosts, attacking them at the roots. Root tips of plants usually manage to escape infection even when directly inoculated with spores of a fungal pathogen. This happens through a process in which the pathogen is prevented from forming intimate contact with the root surface (Gunawardena and Hawes, 2002). Plant roots harbor a mucilaginous matrix or “slime” around them that contains a population of living cells, referred to as “border cells”, which have separated from the root cap into the environment. Border cells bear intact CWs and are metabolically active, yet their metabolism is significantly different from that of the root cap cells they originate from. The export of newly synthesized DNA into the extracellular matrix among detaching cells at the root cap periphery from *Convolvulus arvensis* was first documented in 1971 (Phillips and Torrey, 1971), but at the time its biological significance remained unknown.

Analysis of the extracellular mucilage surrounding border cells confirmed the presence of host exDNA even in the absence of pathogens. Enzymatic digestion of this exDNA using DNase I abolished root tip resistance to infection (Wen et al., 2009). Recently, the delivery of plant exDNA in living border cell populations separated from the root caps of pea and corn plants was visualised for the first time (Wen et al., 2017). This study revealed that exDNA is secreted as new border cells disperse from the root cap periphery and that exDNA plays a critical scaffolding role in the structural integrity of the complex extracellular trap structures surrounding root border cells. Border cells may thus provide a protective role to the root tip meristem against infection and injury to maintain development and ensure survival (Hawes et al., 2011; Wen et al., 2017).

## Taking Secretomics Seriously: Biotechnological Applications to Solve Food Security Constraints

Given the significant economical and societal impact of plant diseases caused by microbial pathogens, molecular components involved in the control of such devastating diseases have been the object of several biotechnological studies and applications, notably genetic improvement of plant resistance. Identification and introgression of resistance traits into desirable varieties through accelerated breeding and gene editing technologies is essential to global food security (Ghag, 2017; Silva et al., 2018; Pixley et al., 2019; Yin and Qiu, 2019).

Owing to their central role in plant-fungi interactions, fungal secreted effectors are being investigated to address their biotechnological potential in agriculture, using the knowledge gained from effector biology in recent years. In a recent review by Van de Wouw et al. (2019), one strategy is referred to as ‘effector-guided’ breeding and involves the use of purified or heterologously-expressed effectors as well as recombinant effector proteins in agroinfiltration experiments for pathogen-resistance screening purpose. Another approach is based on the characterization of effector-induced defense responses, also referred to as effector-triggered immunity, in particular those mediated by a core of conserved effectors that play key roles in disease development. This will allow the identification of interacting resistance genes in host plants, hence hopefully a more durable resistance. Therefore, the identification of candidate plant resistance genes, using effector-triggered immunity, provides high-throughput tools for screening germplasm and breeding material. Given that traditional breeding is a slow process requiring numerous generations of selection, resorting to biotechnology approaches such as genome editing and GM crops can accelerate the crop improvement process. (Van de Wouw and Idnurm, 2019).

The use of natural and engineered EVs in drug delivery has been used to address several human health problems such as cancer, hepatitis C, neurodegenerative diseases, inflammatory states etc. (Saenz-Cuesta et al., 2015; Raimondo et al., 2019; Wiklander et al., 2019). Furthermore, it is surmised that vesicle-mimetic delivery systems offering properties similar to those of natural EVs will bring new opportunities to deliver cargos to a target cell (Garcia-Manrique et al., 2018; Surman et al., 2019). In recent years, particular attention has been drawn to non-animal (especially plant) EVs and their potential use in human therapy. In particular, fruit-derived exosomes have been isolated, characterized and tested as beneficial products (Wang et al., 2014; Raimondo et al., 2015; Zhuang et al., 2016; Garcia-Manrique et al., 2018). Therefore, the possibility of using edible plant-derived vesicles for the loading of other compounds, whether of vegetal or synthetic origin, for therapeutic purposes appears very promising (Rome, 2019), especially considering that plant EVs can be produced in large quantities and at low cost (Raimondo et al., 2019).

Finally, the demonstration that plant EVs isolated from sunflower seedlings are incorporated by the highly invasive fungal pathogen *S. sclerotiorum* and cause fungal growth inhibition (Regente et al., 2017) pave the way to develop plant EVs as a delivery system of drugs (metabolites, proteins, nucleic acids) able to combat bacterial and fungal pathogens, as demonstrated for animal EVs in human therapy (Wiklander et al., 2019).

## Conclusions

Although numerous plant and fungal secretome studies have predominantly focused on protein secretion, the data reported in the present review highlight that both plants and their interacting fungi secrete a cocktail of nonproteinic molecules including metabolites, phytohormones and nucleic acids, thereby unveiling the multiple facets of plant-fungal interactions. Accordingly, we propose to extend the definition of the plant and fungal secretomes to a broader sense that includes both proteinic and non-proteinic secretions, to comprehensively unravel the functioning of the plant/microorganisms holobiont. How all these secreted molecules are orchestrated and how they interact to sculpt and regulate communication mechanisms between plants and fungi, in both beneficial and pathogenic interactions, remain to be elucidated. It will certainly be the object of future studies aiming at improving crop protection and achieving sustainable agriculture, particularly in the context of anticipated climate changes and increasing global food demand.

Plant–microbe interactions have evolved over hundreds of millions of years, generating a diversity of associations covering a broad spectrum from pathogenic to mutualistic coexistence (Martin et al., 2017; Uhse and Djamei, 2018). Although these various lifestyles incur different needs, they all bear in common the use of secreted molecules, which enable interacting partners to communicate and have an impact on each other and on their environment, and *vice versa*. Given that secreted molecules, including secondary metabolites, are critical for intercellular communication, it is likely that both plants and their associated fungi have evolved to adopt identical secretion processes, namely conventional and unconventional mechanisms, allowing them to optimize their mutualistic interactions or to combat and counterattack during pathogenesis.

We anticipate that the development of comprehensive secretome studies in plants and fungi will offer new avenues for the identification of proteinic and nonproteinic molecules that can be exploited to develop novel crop protection strategies, notably through the use of plant EVs in drug delivery. Comprehensive secretome analysis will also allow the characterization of plant genes and metabolites central to plant root-rhizospheric microbiote interactions towards improving plant production and protection, while optimizing the use of agrochemical products.

## Author Contributions

DV, MR, DJ: drafted, edited, and reviewed the manuscript.

## Conflict of Interest

The authors declare that the research was conducted in the absence of any commercial or financial relationships that could be construed as a potential conflict of interest.

## Abbreviations

AA, amino acid; ABA, abscisic acid; ABC, ATP-binding cassette; ALMT, Aluminum-Activated Malate Transporter; AMP, antimicrobial peptide; ATP, adenosine 5′-triphosphate; AVR, avirulence; BBE, berberine bridge enzyme; CAZyme, carbohydrate-active enzyme; CELLOX, cellodextrin oxidase; CK, cytokinin; CLE, CLAVATA3/ESR; CW, cell wall; CWDE, CW degrading enzyme; CWME, CW methylesterase; DAMP, damage-associated molecular pattern; DCL, dicer-like; DNA, deoxyribonucleic acid; DORN1, Does not Respond to Nucleotides 1; eATP, extracellular ATP; ER, endoplasmic reticulum; ERCC, Extracellular RNA Communication Consortium; EV, extracellular vesicle; exDNA, external DNA; exDNase, extracellular DNases; exRNA, extracellular RNA; GA, gibberellin; GMO, genetically-modified (GM) organism; GUS, β-glucuronidase; HIGS, Host-Induced Gene Silencing; IAA, indole-3-acetic acid; iDNA, internal DNA; IM, invasion molecule; IP, invasion patterns; ISR, induced systemic resistance; JA, jasmonic acid; KEP, KEX2-processed repeat protein; LSP, leaderless secretory proteins; MAMP, microbe-associated molecular pattern; MAPK, mitogen-activated protein kinase; MATE, Multidrug and Toxic compound Extrusion; MFS, Major Facilitator Superfamily; miRNA, microRNA; NGS, next generation sequencing; nt, nucleotides; OG, oligogalacturonide; ORF, Open Reading Frame; P2K, P2 receptor kinase; PAMP, pathogen-associated molecular pattern; PG, polygalacturonase; PGPF, plant growth-promoting fungi; PRR, pattern recognition receptor; PKS-NRPS, polyketide synthase-nonribosomal peptide synthetase; PME, pectin methylesterase; PMEI, PME inhibitor; PR, pathogenesis-related; PRR, pattern recognition receptor; PTM, posttranslational modification; RNA, ribonucleic acid; RNAi, RNA interference; ROS, reactive oxygen species; SA, salicylic acid; siRNA, small interfering RNA; SP, signal peptide; sRNA, small RNA; STM, SHOOT MERISTEMLESS; tyRNA, tinyRNAs; UPS, unconventional protein secretory.
